# Supplementary material for: Are carbon emissions trading and green financial instruments synergistic? -Comprehensive quantitative research based on content analysis
Source: PLoS One. 2024 Mar 7;19(3):e0298601. doi: 10.1371/journal.pone.0298601 (PMC10919601; doi:10.1371/journal.pone.0298601)
Supplement: S1 Data — (ZIP) [file pone.0298601.s001.zip › Data/地级市Z_F工作报告.zip!/║■─╧/╘└╤⌠╩╨/└┤╫╘╣1⁄2╓┌║┼í╛┬φ┐╦╩2╛▌═°í┐_2017.docx]

各位代表：

现在，我代表市人民政府，向大会报告政府工作，请予审议，并请市政协委员和其他列席人员提出意见。

岳阳发展站上了新的更高起点

过去四年，在中共岳阳市委的坚强领导下，我们全面贯彻党中央、国务院和省委、省政府各项决策部署，团结拼搏，克难奋进，较好完成了本届政府工作任务，岳阳发展站上了新的更高起点。

经济走势更加强劲。四年来，我们顶住宏观经济下行压力，全力以赴稳增长，保持了综合实力全省第二的位置。全市GDP迈上3000亿元台阶，由2012年的2199.9亿元增加到2016年的3123亿元（预计数，下同），年均增长9%；固定资产投资、规模工业增加值、社会消费品零售总额、公共财政预算收入分别达到2307亿元、1110亿元、1143亿元和330.8亿元，年均分别增长20.6%、9%、12.8%和9.4%，主要指标增幅均居全省前列。产业结构不断优化，三次产业结构比为10:48:42。工业结构进一步转优，形成了石化、食品两大千亿产业集群和11个省级以上产业园区，高新技术产业、非公工业占规模工业比重分别达到19.5%和80%。第三产业贡献加大，占GDP比重比2012年提高9.2个百分点；成功组建4家农商行，引进5家股份制商业银行。现代农业加快发展，新增市级以上农业产业化龙头企业60家，亿元以上农产品加工企业发展到153家，粮食产量稳定在310万吨以上。

改革开放更加深入。10大类155项改革稳步推进，供给侧结构性改革、行政审批制度改革、不动产统一登记制度、PPP模式试点等走在全省前列；营改增、公车改革、国企改革、城市公立医院改革等达到预期目标；政府机构改革、乡镇区划调整和并村工作等全面完成。对外开放取得重大突破，发扬“挑战不可能”的精神，经过多年持之以恒的努力，“一区一港四口岸”成功获批并建成运营，岳阳成为全国唯一拥有6个国家级口岸平台的地级市；成功组建湖南城陵矶国际港务集团；开通直达、接力航线4条，2016年港口集装箱吞吐量达到29.4万标箱，进出口总额比2012年翻了一番。

城乡面貌更加秀美。基础设施建设步伐加快，四年实施亿元以上重大交通项目51个，新建高速公路5条254公里、干线公路29条635公里，随岳、通平、岳常、石华、大岳高速和芙蓉大道湘阴段等建成通车；完成各类水利工程22.3万处，除险加固水库574座，治理中小河流253公里；投产输变电项目57个，升级改造农网1776个村，完成智能化用电改造80万户。城市品质不断提升，中心城区控详规划基本实现全覆盖，常住人口城镇化率达55.5%。四年实施亿元以上重大城建项目71个，中心城区重点渍水地段整治、小街巷改造、道路交通微循环改造三个“三年行动计划”全面完成，王家河流域综合治理等扎实推进，环南湖周边亮化工程和南湖公园、金鹗公园提质改造如期竣工，成为市民休闲健身的好地方。生态环境加快改善，湘江、洞庭湖、南湖水环境治理和铁山水资源保护持续加力，农村环境综合整治深入推进，森林覆盖率和空气质量优良率分别达45.3%和77.3%。岳阳获评全国文明城市、全国宜居城市、中国十大活力休闲城市等荣誉称号，城乡美景新貌刷爆“朋友圈”，广大群众纷纷点赞。

人民生活更加幸福。四年累计民生支出突破1000亿元，占财政支出比重72.8%；城乡居民人均可支配收入分别达27521元和13143元，比2012年分别增加8220元和4386元；全面小康实现程度达94%。扶贫攻坚成效显著，贫困发生率下降至6%。社会保障扩面提标，“五险”参保人数和征缴基金稳步增长；城乡低保月补差分别达300元和130元；残疾人“两项补贴”政策惠及5.6万人；新增城镇就业22.3万人、农村劳动力转移就业23.8万人，“零就业”家庭实现动态清零。住房保障力度加大，建成各类保障性住房17.6万套。社会事业繁荣发展，率先全省实行阳光招生，新建和改扩建城镇学校62所，建成义务教育合格学校488所、农村公办幼儿园173所；大力实施巴陵人才工程和“四海揽才”计划，培育并引进各类人才2万多人；完成县级医院、乡镇卫生院改扩建项目133个，救助大病患者7200人；县级社会福利中心覆盖率达100%；全民健身设施覆盖率达84.8%。更新新能源公交车513辆，建成公共自行车租赁系统，绿色出行成为新风尚。

民主法制更加健全。坚持依法治市，出台《法治岳阳建设纲要》和法治政府建设实施方案，为行政行为划定更加严格的法治轨道。自觉接受市人大法律监督和市政协民主监督，承办人大议案、代表建议和政协建议案、委员提案2171件，办结率、满意率分别达100%和99%，“美好社区、温馨家园”议案办理取得好的成效，岳阳楼区获评全国和谐社区建设示范城区、云溪安居园等4个社区分别获评示范社区。深入开展“三提升”主题活动，率先全省不再保留非行政许可审批事项，行政审批提速43.8%。创新社会治理与服务“三合一”模式，岳阳楼区下放公共服务事项111项。全面落实党风廉洁建设“一岗双责”，深入开展党的群众路线教育实践活动、“三严三实”专题教育和“两学一做”学习教育，严格落实中央“八项规定”精神，政风行风持续好转。

刚刚过去的2016年，我们按照中央和省委、省政府的工作部署与要求，扭住发展第一要务，聚焦重点，精准发力，经济社会保持平稳健康发展。预计全市GDP增长8%，规模工业增加值增长7%，固定资产投资增长14.5%，社会消费品零售总额增长12%，公共财政预算收入增长9.2%，居民人均可支配收入增长9.3%，主要经济指标增幅稳居全省第一方阵。

一年来，我们突出抓大事、要事、实事、难事，围绕推进“一极三宜”江湖名城建设，一以贯之抓落实，实现了“十三五”良好开局。

一批重大项目实现了快速推进。发挥投资对稳增长的关键作用，牢牢抓住项目建设这个“牛鼻子”，以大项目带动大投资，以大投资推动大发展。项目建设速度进一步加快。举办四次重大项目限时开工和集中开工竣工，开工项目124个，竣工项目51个，涉及总投资665.6亿元，掀起了项目建设的新高潮，省级主要新闻媒体多次重点推介，产生了很好的示范效应。“双百工程”攻坚提速，107个重大在建项目完成投资489.8亿元，占年计划的108.7%，103个重大前期项目推进顺利。京港澳高速新开联络线、S308平江段大修工程等全面建成；三荷机场、蒙华铁路岳阳段、学院路综合提质改造等扎实推进；500千伏岳阳南输变电工程建成投产。干成了一批多年想干的项目，环南湖旅游交通三圈提前2年全线拉通，临湖公路、G240岳阳县至湘阴段改扩建工程等一批谋划多年的项目成功实施，一批进展缓慢的项目全面提速。项目招商质量进一步提高。全市内联引资项目480个，南翔万商国际商贸物流城一期、岳阳现代装备制造产业园一期等竣工运营，友阿商业城市综合体、金悦洋商业公园、海济生物等一批投资过10亿元项目成功落地；5000万元以上项目增加到740个，224个项目纳入省级年度重点投资计划。项目融资渠道进一步拓展。设立100亿元城市建设发展基金，有效撬动了一批城建项目加快实施；推广PPP模式，21个项目进入国家级和省级示范项目笼子，项目数量和投资总额均居全省前列。这些重大项目的实施，既有力拉动了当前经济增长，又为岳阳长远发展提供了强力支撑。

一批创新工作进行了有益探索。坚持以创新的举措推动发展，在工业园区、县域经济、“三农”工作等方面解放思想、大胆探索，取得了好的成效。狠抓工业园区发展。牢固树立“实力看工业、工业看园区”的工作理念，出台“园区十条”，这是我市近年来首个专门支持园区发展的文件，在园区审批、用人和分配等机制上大胆创新，重点对城陵矶新港区、经开区分别下放41项、48项审批权限，为园区发展减压松绑，赋予了园区更多自主权。园区工业增加值占规模工业比重达67%，同比提高6.9个百分点，3个园区工业增加值增速排名全省前10位；强力推进“135”工程建设，竣工标准化厂房268万平方米，新增创新创业企业419家，超额完成省定目标任务。狠抓县域经济发展。牢固树立“强市必先强县”的工作理念，明确县域经济“六个一”工作重点，持续开展“学赶超”活动，7个县市区财政收入过10亿元，3个县市区综合实力进入全省20强。狠抓农业农村发展，推进工商资本下乡，流转农村耕地182.6万亩，培育新型农业主体39620个；启动屈原农村改革集中区创建工作，这是全省第一个农村改革集中区，通过全面推进“六化”，致力率先全省探索一条破解“三农”难题的新路子。创新已成为我市破解发展瓶颈的“金钥匙”，推动经济增长的“新动能”，成为岳阳加快建设湖南发展新增长极的强大引擎！

一批优势产业保持了稳健发展。坚持新兴产业培育与传统产业提质“两手抓”，重点扶持9大优势产业发展。做长石化产业链条。建立厂地联席会议和规范对接机制，完善绿色化工产业园区规划，举办石化产业招商推介会，签约项目总投资达20亿元，致力将绿色化工产业园建设成为全省最有特色的现代产业园区。做旺旅游市场人气。强力推进南湖13118工程、洋沙湖国际旅游度假区等项目建设，成功举办首届岳阳洞庭湖生态旅游文化节、岳阳市民免费游岳阳等活动，旅游总人数、总收入继续保持高位增长，岳阳大酒店获评我市第一家五星级旅游饭店，实现了全市五星级酒店“零”的突破。做大现代物流规模。综合保税区封关运行；岳阳新港首次实现盈利，扭转了6年来连续亏损的局面；55家外贸企业“破零倍增”；岳阳获批全国现代物流创新发展试点城市。 其他优势产业持稳发展，食品加工、生物医药、装备制造、军民融合、清洁能源、节能环保等进一步扩张，产业基础更加坚实。

一批民生实事取得了显著成效。坚持把“两改”作为改善民生的重要抓手，组织实施我市近年来规模最大、力度最大的城市棚户（旧城）区改造和农村危（旧）房改造，完成城市棚户区改造31107户，中心城区消化商品房库存114万平方米。中心城区“十大片区”改造计划征收房屋200万平方米，拉动投资1000亿元，出台6条拆迁奖励政策，惠及1.2万户近5万人，目前这项工作进展顺利，基本完成征拆协议签约。在拆迁任务空前繁重的情况下，没有发生一起非正常群体性上访事件，这是很不容易的。推动农村危（旧）房改造与规范村民建房相结合，规划建设255个集中建房点，已开工82个，审批建房10978户，拉动民间投资20.7亿元。我市“两房两棚”工作得到省委、省政府的高度肯定，汨罗南托新村示范点经验被省委《工作情况交流》重点推介。强力推进精准扶贫“三年行动计划”，全市组建344个扶贫工作组，实施贫困户帮扶项目3.27万个，77个易地扶贫搬迁项目全部开工，8.77万贫困人口成功脱贫。统筹推进其他民生事业发展，中心城区学校“六建六扩”步伐加快，市十四中完成置换搬迁，市十六中主体工程竣工，顺利通过国家卫生城市复查，成功创建国家公共文化服务体系示范区，26项37件省市实事全面完成。岳阳进入中国地级市民生发展百强，居第58位。

一批发展难题得到了有效破解。我们坚持问题导向，聚焦发展难题，找准“症结”，开好“药方”，实实在在推动当前工作和长远发展。着力破解生态环境难题。启动湘江保护和治理第二个“三年行动计划”、洞庭湖水环境综合整治“五大专项行动”，推行常态化、长效化监管整治机制，采砂总量、运砂船舶分别减少40%和30.7%，特别是七里山水域整治成效明显，运砂船舶锚泊总量大幅减少，水域通航及停泊秩序有效改观，得到省委主要领导的充分肯定；加强南湖、王家河等城市水系治理和农村环境综合整治，“水墨丹青”底色更加厚重，岳阳获评“2016中国十佳绿色城市”。着力破解征地拆迁难题。保持禁拆治违和土地依法征收“两大行动”高压态势，中心城区拆除违建5296处110.5万平方米，拆违面积是2015年的近2倍，土地依法征收清零项目86个、腾地17837亩，是我市项目用地保障力度最大的一年。着力破解社会治理难题。推行“大部门、大警种”警务机制改革和火车站片区综合执法，全国公安机关规范基层警务工作暨110接处警工作会议在我市召开，岳阳经验在全国推介。深入开展“安全监管执法年”、安全生产“大排查大管控大整治”专项行动，强力推进路域环境综合整治和治理超载超限，认真抓好信访维稳和“平安岳阳”创建工作，综治工作荣获全省先进，社会大局和谐稳定。

其他事业协调发展，通过加强国防动员、武警、消防、人防等工作，开创了军政军民团结新局面；通过加强统计、史志、档案、国安、保密、老干老龄、民宗、科协、气象、联络接待、公积金、移民、烟草、盐业、邮政、通信等工作，推动了各项工作齐头并进；通过支持工会、共青团、妇联、工商联及各民主党派、无党派人士等工作，凝聚了新增长极建设的强大合力。

各位代表！过去四年是我市历经考验、攻坚克难的四年，也是抢抓机遇、奋勇争先的四年。成绩来之不易，经验弥足珍贵！这是省委、省政府和市委坚强领导的结果，是市人大、市政协监督支持的结果，是全市上下共同努力的结果。在此，我代表市人民政府，向全市人民，向所有关心支持岳阳发展的社会各界人士，表示衷心的感谢和崇高的敬意！

奋斗尤为艰辛，发展时不我待。我们清醒认识到，岳阳发展还面临着不少困难和问题：一是经济下行压力仍然存在，经济总量还不够大、质量还不够优、效益还不够高，向中高端水平迈进任重道远；二是产业结构性矛盾仍然突出，县域经济、开放型经济、金融经济等短板制约明显，结构调整任务繁重；三是城乡发展差距仍然较大，基础设施、社会保障、公共服务体系建设还不平衡，城乡统筹发展任务艰巨；四是干部作风建设仍然有待加强，反“四风”、转作风还需要进一步从严从实，等等。我们一定要正视这些问题，不能回避这些问题，在今后工作中采取强有力的措施，认真加以解决。

加速加力推进新增长极建设

打造湖南发展新增长极是省委、省政府对岳阳发展的新定位、新要求，也是全市人民的新愿景、新梦想，把岳阳摆到了前所未有的战略高度，赋予了岳阳前所未有的历史重任，我们义不容辞、责无旁贷。时代呼唤作为，使命催人奋进！岳阳作为排名全省第二的经济强市、全省唯一通江达海的港口城市、“一带一部”的重要节点城市，我们务必顺应时代发展要求和群众美好期待，集全市之智，举全市之力，加快推进新增长极建设，奋力推动宏伟蓝图变成美好现实。

实现宏伟蓝图，需要我们精准研判发展形势。今后五年，我市面临着宏观经济下行和转型升级的双重压力，但经济长期向好的基本面没有改变，洞庭湖生态经济区、长江经济带、长江中游城市群和湖南“一核三极四带多点”区域布局等重大战略全面实施，给岳阳带来了千载难逢的政策机遇；特别是通过历届市委、市政府矢志不渝的努力，我市构建了更加坚实的基础设施体系、更为完善的产业园区体系、更具优势的开放平台体系、更有活力的机制创新体系，为岳阳未来发展打下了良好基础，积蓄了强大后劲，有利于我市在新的起点上实现新的跨越。岳阳“一极三宜”江湖名城建设其时已至、其势已成，必将迎来一个大有希望、大有作为的黄金期。我们要进一步增强机遇意识，用好用活政策机遇，化压力为动力，化挑战为机遇，将新增长极建设奋力推向前进。

实现宏伟蓝图，需要我们科学确定奋斗目标。目标是我们坚定前行的动力，是我们工作努力的方向，目标明则思路清、方向准、步子稳。今后五年政府工作总体要求是：高举中国特色社会主义伟大旗帜，深入学习贯彻习近平总书记系列重要讲话精神，全面落实党的十八届六中全会和省第十一次党代会、市第七次党代会精神，按照“五位一体”总体布局和“四个全面”战略布局，积极践行“五大发展”理念，大力实施“创新引领、开放崛起”战略，加快建设“一极三宜”江湖名城，奋力谱写富饶美丽幸福新湖南的岳阳篇章!

今后五年的主要目标是：

——综合竞争实力明显增强。力争到2021年，全市GDP迈上5000亿元台阶，人均GDP达到10000美元以上。固定资产投资、社会消费品零售总额、公共财政预算收入分别迈上5000亿元、2000亿元、500亿元台阶。主要经济指标占全省比重达到12%左右。

——经济质量效益明显提高。三次产业结构比进一步优化。园区工业占规模工业增加值比重达到70%以上。科技进步贡献率达到60%以上，高新技术产业和民营经济占GDP比重分别达到23%和67%。进出口总额突破50亿美元，集装箱吞吐量达到40万标箱。

——城乡发展环境明显改善。全市常住人口、户籍人口城镇化率分别达到60%和40%。万元GDP能耗下降15%，空气质量优良率、森林覆盖率保持在83%、45.3%以上，Ⅲ类以上地表水水体比例达到98.3%，建成美丽乡村200个以上。

——人民幸福指数明显提升。居民人均可支配收入突破3万元，城镇登记失业率控制在4%以内，市场物价指数稳定在合理区间，现行标准下贫困人口全面脱贫，让广大群众共同享有人生出彩、梦想成真的机会。

——社会文明程度明显进步。社会主义核心价值观深入人心，全民素质显著提升，法治岳阳、平安岳阳、诚信岳阳建设取得新成效，社会安全指数保持100%，文明城市指数测评保持全省先进，社会大局和谐稳定。

实现宏伟蓝图，需要我们切实找准工作抓手。当前，岳阳最大的问题还是发展的问题，还是经济总量不大不强、经济发展不快不优的问题。我们要准确把握政府工作要求，切实强化目标导向，以踏石留印、抓铁有痕的决心，推进新增长极建设，坚决做到不达目的不罢休、不获全胜不收兵。我们要牢牢把握岳阳经济新方位，主动适应把握引领经济发展新常态，把发展作为解决一切问题的“总钥匙”，自觉遵循发展的基本规律、基本原则和基本要求，多出实招，能出新招，敢出硬招，努力创造经得起实践检验、人民检验和历史检验的工作业绩。必须坚持创新驱动。始终把创新作为推动发展的核心动力，以科技创新为引领，推进观念创新、制度创新、文化创新等各方面的全面创新，形成创新主体充满活力、创新链条有机衔接、创新治理更加科学、创新效率大幅提高、科技文化与经济发展高度融合的创新新格局，综合创新能力处于全省第一方阵，推动经济增长由投资驱动向创新驱动加速转变。必须坚持对外开放。始终把开放作为岳阳崛起的战略支撑，以融入“一带一路”为切入点，坚持“引进来”与“走出去”并重、对外开放与对内开放并重、引资引技引智与优化发展环境并重，实现更宽领域、更深层次、更高水平的开放，把岳阳打造成为内陆对外开放的新高地、国际物资集聚的新基地。必须坚持生态优先。始终把生态文明建设贯穿发展的全过程，树立绿水青山就是“金山银山”的强烈意识，决不以牺牲环境为代价换取一时的经济增长。科学划定生态红线，实施循环发展引领计划，强力推进大气、水、土壤三大污染防治行动，加快建设蓝天常驻、青山常绿、碧水常流的美丽岳阳。必须坚持民生为本。始终把“以人民为中心”作为政府工作的出发点和落脚点，加大财政民生支出力度，扎扎实实办好每一件民生实事，统筹解决群众广泛关注的社会就业、社会保障、教育医疗、扶贫帮困、危房改造等问题，确保人民群众获得感、幸福感明显提升，归属感、认同感明显增强。必须坚持求真务实。始终把“一切从实际出发”作为政府工作基本要求，决不搞脱离实际、沽名钓誉的政绩工程，决不搞劳民伤财、华而不实的形象工程，决不搞失真失实、虚报浮夸的水分工程，一步一个脚印抓发展，努力追求真金白银、实实在在的政绩。“五个必须坚持”是今后五年政府工作的基本遵循，我们将以“功成不必在我，建功必须有我”的责任和担当，全力推进新增长极建设，以卓越的工作业绩向建党一百周年献礼！

率先环洞庭湖地区全面建成小康社会

2017年是新一届政府开局之年，是我市全面建成小康社会决胜之年。面对艰难繁重的发展任务和人民群众的美好期盼，我们将认真贯彻落实中央、省委和市委经济工作会议精神，坚持稳中求进工作总基调，全面做好稳增长、促改革、调结构、惠民生、防风险各项工作。主要预期目标是：GDP增长9%左右，规模工业增加值增长8%，固定资产投资增长16%，社会消费品零售总额增长12.5%，公共财政预算收入增长10%左右，居民人均可支配收入增长9%，率先环洞庭湖地区全面建成小康社会。主要任务是：

一、狠抓产业结构调整，增创发展新优势

岳阳产业结构还不够优、层次还不够高，必须大力推进产业转型升级，加快构建多点支撑、集群发展的现代产业体系，进一步提高发展质量和效益。

提升工业发展水平。新增长极建设必须要有大企业、大工业作支撑，否则就是一句空话。我们将始终坚持工业强市、园区兴工和项目立园，紧紧围绕工业园区、优势产业、骨干企业谋划工业发展，加快打造岳阳工业转型发展的升级版。2017年，全市要新增规模工业企业80家以上，园区工业占规模工业比重提高2—3个百分点。全面落实市委、市政府已经出台的“园区十条”，推动项目向园区集中、产业向园区集聚，引导每个省级以上园区重点发展2—3个主导产业，坚定不移走特色化、集约化的发展路子。全面壮大优势产业，每个产业筛选确定3—5家核心企业，通过延伸优势产业链条，扩张核心企业规模。进一步做大做强石油化工、食品加工2个千亿产业集群，改造提升清洁能源、装备制造等传统产业集群，培育壮大军民融合、生物医药、节能环保等新兴产业集群。严格落实县域经济“六个一”工作重点，将县域经济发展聚焦到园区、产业和项目上来，引导各县市区培育做大1—2个优势产业。全面推进企业帮扶，实施重点企业“一企一策”，研究出台规范企业收费的文件和政策，扎实开展降成本专项行动，着力解决融资、用地、审批、环境等难题，一心一意为企业排忧解难，全心全意为企业保驾护航，让企业卸下包袱、轻装上阵、企稳前行，走得更快更稳、更远更好！

补齐三产发展短板。坚定不移推进消费升温升级，进一步扩大新兴消费、稳定传统消费、挖掘潜在消费，依靠消费助力经济发展。坚持把旅游业作为第三产业的龙头来抓，以打造古城旅游、江湖旅游、山岳旅游三大板块为重点，突出抓好3517文旅城、洞庭湖博物馆、天岳幕阜山、汨罗江端午文化产业整体开发等旅游综合体建设，加快创建平江、临湘、湘阴三个国家全域旅游示范区，办好特色旅游节会活动，致力打造旅游目的地城市。当前，就我市旅游业发展现状来看，体制机制创新是必要的，是推动旅游业跨越发展的正确选择。今年，我们要大力引进战略投资者，加大景区体制机制创新力度，着力补齐旅游业发展短板，尽快把旅游业打造成为支柱产业。我市物流业发展潜力巨大、前景广阔，我们将专门研究出台支持物流业发展的具体办法，着力扶持物流龙头企业发展，致力把物流业打造成为千亿产业。优化中心城区商贸布局，加快友阿商业城市综合体、红星美凯龙和专业批发市场建设，加大五星级酒店引进力度，逐步做大东茅岭、花板桥、南湖片区、高铁新城等城市商圈。积极化解房地产库存，促进房地产市场平稳健康发展。

转变农业发展方式。把推进农业供给侧结构性改革作为农业农村工作的主线，以增加农民收入为主要目标，持续提高农业效益和竞争力。大力发展现代农业，突出抓好华容和屈原国家现代农业示范区、洞庭湖绿色食品产业园、岳阳黄茶标准化产业园、台湾农民创业园等农业园区建设，重点打造“一个300万亩、三个50万亩”特色生产基地。实施最严格的耕地保护制度，确保耕地数量和质量占补平衡，粮食播种面积稳定在800万亩以上。严格落实“三权分置”农村土地管理制度，促进土地有序流转，鼓励更多工商资本下乡，大力培育农业种养大户、家庭农场、专业合作社、龙头企业、农村电商等经营主体，盘活农村资产资源，推动农业适度规模经营，让农业真正成为有潜力、有希望、有奔头的朝阳产业、富民产业！

二、狠抓基础设施建设，强化发展新支撑

基础设施建设是扩大有效投资的重要载体，我市还有很大的提升空间，必须坚持不懈地抓下去，加快提升发展承载能力。

构建互联互通的交通体系。一条路往往可以改变一个地方的发展格局，助推一个地方的经济腾飞。我们要牢固树立“交通先行”的发展理念，全力打造区域性综合交通枢纽城市。今年底要完成三荷机场主体工程，基本具备通航条件，规划建设航空口岸和空港产业区。扎实推进蒙华铁路、华容监利长江公铁两用桥等项目建设，稳步推进疏港铁路前期工作。加强长江、湘江航道岳阳段疏浚治理和锚地建设，进一步提升通航能力。临湖公路、赶山东路、金凤桥北路、G240岳阳县至湘阴公路要在年内建成通车。加快平益高速、岳望高速、岳阳东客运枢纽、高铁东站至三荷机场快速通道建设，规划实施环洞庭湖路、G107岳阳段东移暨升级改造、S306岳阳段升级改造、汨罗至杨桥公路升级改造、岳阳长江经济带沿江公路（黄盖湖至道仁矶）、中心城区西环线、S316改造升级等项目。要通过推进交通大建设，实施交通大会战，全面优化路网结构，加快打造外部配套成环、内部纵横成网、铁公水空“四位一体”的大交通格局，为岳阳发展插上腾飞的翅膀。

构建保障有力的水利体系。水情是岳阳最大的市情，必须把兴水利、治水患摆在更加重要的位置，全面推进人水协调的现代水利体系建设。聚焦“4830”目标，突出抓好“十大工程”，完成水毁工程修复4700处，清淤整治渠道5000公里，加固堤防300公里，治理水土流失面积51.6平方公里、河道33.9公里，加强防洪抗旱设施建设和山区地质灾害防治，完成水库除险加固、小型农田水利建设、农饮巩固提升工程、水利“百千万”工程等年度建设任务。大力发展民生水利、安全水利、生态水利和资源水利，坚决打赢水利建设“攻坚战”，坚决守住农业发展“命根子”！

构建绿色安全的能源体系。坚持绿色发展，推动能源生产和消费革命，促进能源结构优化升级。加快推进华电平江电厂、神华国华岳阳电厂、静脉产业园、福寿山抽水蓄能电站等项目的相关工作。扎实抓好新一轮农网升级改造，竣工投产输变电项目12个，开工建设农村水电增效扩容改造项目12个，实现智能化用电改造全覆盖。加快“气化湖南”支线管道工程和重点乡镇天然气管线建设，逐步实现重点乡镇燃气管道化。

三、狠抓深化改革创新，释放发展新活力

当前，改革已进入了攻坚期，再难也要向前推进。全市上下要以改革的举措、创新的思维开创发展新局面，努力谋求发展的“最大公约数”。

突出深化改革。以供给侧结构性改革为主线，全面落实“三去一降一补”五大任务，努力破解发展的体制机制障碍。深化行政体制改革，上半年要完成市本级和临湘、华容承担行政职能事业单位改革试点；启动经营类事业单位改革和行政综合执法体制改革；继续精简行政审批事项，持续提高行政效能。深化经济体制改革，抓好国企“三供一业”分离移交工作；加大各领域PPP模式推广和监管力度，杜绝固定回报、明股实债的“伪PPP”项目；深化预算管理、预决算公开、债务管理改革，积极防范和化解政府债务风险；发展多层次资本市场，大力引进股份制商业银行和村镇银行，拓宽保险、证券服务领域，引导民间资本稳妥有序参与金融市场。深化农村综合改革，扎实抓好合乡并村后续工作，完成农村土地确权登记颁证，强力推进屈原农村改革集中区创建工作，大胆探索“乡建投”“土地超市”“家庭联盟”等具有本地特点的农村改革新举措，力争率先全省出成果、出经验、出典型。

突出科技创新。实施创新驱动战略，最根本的是要增强自主创新能力。今年要把人才工作摆到更加重要的战略位置，继续推进巴陵人才工程，切实加大人才引进、培养、使用和激励力度。健全完善院士工作站、重点实验室、工程研究中心、企业孵化器等科技创新平台体系。以发展新技术、新产业、新业态为重点，围绕优势产业开展关键技术攻关和新产品研发，促进科技成果转化，新增规模以上高新技术企业15家以上。

突出全民创业。就业是民生之本，创业是就业之源。今年要大力实施“创新创业引领计划”和“创业带动就业促进计划”，全面落实“百优资助工程”。拓宽创新创业领域，坚持“非禁即入”，放宽政策，降低门槛，鼓励支持非公企业“二次创业”，引导更多社会资本投向实体经济。培育壮大创业主体，大力发展非公经济，重点针对高校毕业生自主创业、寓外乡友返乡创业和外来资本进驻创业，发展一批众创空间和“星创天地”。积极开展“创新创业、先锋先行”活动，弘扬“工匠精神”，让广大创业者敢创业、能创业、创大业，在巴陵大地广泛掀起“精英创业”“大众创业”“草根创业”百花齐放的新浪潮！

四、狠抓对外开放合作，拓展发展新空间

开放只有进行时，没有完成时。岳阳作为首批沿江对外开放城市，必须敞开门户促发展，着力打造湖南对外开放的“桥头堡”，努力昂起融入长江经济带的“大龙头”，奋力争当全省开放崛起的“排头兵”。

加快港区建设。城陵矶港是岳阳开放发展的“黄金资源”，也是岳阳在全省独一无二的禀赋优势，必须坚持以城陵矶新港区为依托，积极融入“一带一路”建设，推动我市外向型经济提速发展。要认真落实省政府《关于支持岳阳城陵矶港建设发展的会议纪要》和我市开放型经济发展“1+2”政策组合文件精神。突出抓好城陵矶新港二期、7个重点码头等港口码头群建设，杭瑞高速冷水铺互通连接线、水空联运快速通道等集疏运体系建设，海吉星农产品物流园、国际物流和加工产业园等园区建设。优化整合码头岸线资源，筹备开通城陵矶至日本、台湾、韩国等近海直达航线。深化与上港集团、宁波港合作和大通关协作，推进关检合作“三个一”通关改革和区域通关通检一体化，建成运营电子口岸和进出口交易中心，打通货物通关的“快车道”。

发挥平台效益。依托“一区一港四口岸”平台优势，争取岳阳进入湖南自贸试验区笼子。健全完善综合保税区功能，最大限度地将平台优势转化为开放效益。积极融入“丝绸之路”经济带建设，争取设立湘欧国际货运班列站点。扎实推进现代物流创新发展城市试点，构建互联网物流公共信息服务平台，引导企业货物从城陵矶港进出，年内力争集装箱吞吐量达到36万标箱。

大力招商引资。积极推进招大引强，鼓励中省企业、跨国公司、大型企业集团在我市设立区域总部、研发中心、生产基地和商贸平台。大力实施精准招商，年内举办2—3次重点产业、重点园区招商推介会。支持优势产业和骨干企业对外扩张，加快拓展国际市场。努力营造宽松透明的政策环境、高效便捷的政务环境、公正公平的法治环境、规范有序的市场环境，真心实意为来岳阳的投资客商抓好协调、搞好服务，让他们真正感受到岳阳是一块投资的沃土、创业的乐土，全面构建“亲”“清”新型政商关系。

五、狠抓城乡区域统筹，构建发展新格局

统筹城乡发展是现阶段的一项重要任务，我们既要激发城市发展活力，更要深挖农村发展潜力，坚定不移走以城带乡、城乡互动的发展路子。

提升中心城区品质品位。全面落实“一个尊重、五个统筹”城市发展要求，做大做美岳阳城区。推进多规合一，严格规划约束，继续开展禁拆治违和土地依法征收行动，科学管好用好每一寸土地，为城市发展预留更多的空间。强力推进中心城区“十大片区”改造，抓好规划、招商、建设等工作，力争两年完成改造任务，把“十大片区”打造成为改善人居环境的样板区、提升城市品位的精品区、拉动投资消费的示范区。加快南湖天灯咀、刘山庙、高铁新城等片区开发，完成市体育中心建设。探索城市建设运营新模式，深入推进海绵城市、地下综合管廊、城市黑臭水体治理“三大试点”。加强交通秩序整治，实施中心城区道路微循环改造第二个“三年行动计划”，认真解决城市交通拥堵和停车难问题。科学编制城市水系规划和“四湖两河”治理规划，加快南湖截污管网建设，启动蛇皮套治理和“三湖”治理工程，建设麻布山省级森林公园，留住城市可持续发展的生态“基因”。

推进县城和小城镇扩容提质。县城是一个地方的“窗口”和“门面”。当前，我市有的县城建设还比较滞后，档次还不高，形象还不优。我们要坚持规划与建设、扩容与提质并重，科学编制县城规划体系，每个县城既要适度拓展新城区，每年又要有计划地改造1—2条老街道。坚持把城镇作为统筹城乡发展的重要节点来抓，按照小而全、小而专、小而特、小而富、小而美的方向，每个县重点抓好2—3个小城镇建设，打造一批功能集中、产业繁荣、特色鲜明的小城镇。促进有能力在城镇稳定就业和生活的常住人口有序实现市民化，让广大群众共享新型城镇化发展成果。

加快美丽乡村建设。把规范村民建房作为美丽乡村建设的突破口来抓，将其与农村危（旧）房改造有机结合、统筹推进。坚持面上“两鼓励两禁止”、点上“五有三到场”的工作思路，强力推进22个市级试点乡镇和255个规划集中建房点建设，年内完成农村危房改造2万户以上。加大农村公路提质改造和管护力度。持续开展农村环境综合整治，加快实施农村清洁工程，推动“美丽岳阳”建设走进农村、走进千家万户。

六、狠抓保障改善民生，共享发展新成果

改善民生是最大的政绩。任何时候，我们都要带着感情、带着责任，倾尽全力做好富民、惠民、安民、乐民之事，努力把经济总量、财政增量转化为老百姓实实在在的生活质量，让幸福暖流在巴陵大地上充分涌流！

推进脱贫攻坚。把精准脱贫作为第一民生工程来抓，以平江山区和铁山库区为“主战场”，坚决打赢整体脱贫攻坚战，确保完成10.08万建档立卡贫困人口脱贫任务。严格落实中央“四个切实”“五个一批”“六个精准”等扶贫工作要求，以“四跟四走”产业扶贫和易地扶贫搬迁为重点，统筹抓好发展生产、公共服务、全民教育“三件大事”，推进水、电、路、业、房和环境整治“六到农家”，在扶贫政策、项目、资金上收拢“五指”，集中力量抓好一批群众看得见、摸得着的扶贫工程，激发贫困群众脱贫致富的内生动力，确保在脱贫攻坚道路上，不落下一个贫困地区、不落下一个贫困群众!

加强环境保护。良好的生态环境本身就是生产力，也是一个地方的核心竞争力。要实施最严格的水资源管理制度，今年起要全面推行“河长制”，认真抓好省“一号重点工程”贯彻与落实，深入实施湘江保护和治理第二个“三年行动计划”，积极开展洞庭湖水环境综合整治“五大专项行动”，巩固提升七里山等重点水域整治成效；切实抓好松阳湖、黄盖湖、汨罗江、新墙河、华容河等河湖综合治理和铁山水库等饮用水源地保护；抓好东洞庭湖湿地保护，提升生态涵养功能；加强农村面源污染治理，擦亮“宜居岳阳”的靓丽名片。对一切破坏生态环境的人和事，动真格、出重拳，不管涉及到谁，一律坚决查处、决不姑息，把好山好水好风光永远留在巴陵大地，永远留给子孙后代！

繁荣社会事业。坚持教育优先，落实乡村教师支持计划，加快农村薄弱学校建设，继续推进中心城区学校“六建六扩”，促进民办教育健康发展，推动高等教育、职业教育开展产教融合，发展更高质量、更加公平的教育。加快建设健康岳阳，坚持计划生育基本国策，统筹推进市县公立医院改革和分级诊疗制度，认真研究中心城区医疗资源布局，突出抓好市中心医院、市中医院、市妇女儿童医院、市公共卫生应急救援指挥中心、岳阳首尔健康城建设，着力解决群众看病难、看病贵的问题。开展全民健身活动，年内举办环洞庭湖国际公路自行车赛。完善公共文化服务设施，加强历史文化名城、名楼、名镇、名村保护，构建覆盖城乡、惠及全民的现代公共文化服务体系，提升岳阳文化软实力。

办好民生实事。积极扩大社会就业，2017年力争新增城镇就业5.2万人、创业主体2.8万个，城镇登记失业率控制在4.1%以内。织密扎牢社会保障网，推进社会保险扩面提标，深化机关事业单位养老保险制度改革，稳步提高城乡低保人均补助标准。加强住房保障工作，完成棚户区改造3万户。大力发展慈善事业。加快完善养老服务中心、老年人活动中心等老年服务体系。力争基层综合文化服务中心覆盖率达到80%以上。完成302个行政村配电网改造。新增管输天然气用户2.8万户。新建和标准化改造农贸市场10家。建成运营智能交通指挥中心。实施“质量强市”战略，强化食品药品安全监管，营造放心消费的市场环境。

维护社会和谐。强化“抓稳定就是抓发展”的工作理念，加快创建“平安岳阳”。推广岳阳楼区社会治理服务“三合一”模式，把政府建到群众“家门口”。严格落实安全生产党政同责、一岗双责、失职追责，把重大风险隐患当成事故来对待，严厉打非治违，确保不发生重特大安全事故。加强双拥共建和国防后备力量建设。改善金融生态环境，维护金融稳定。深化信访工作制度改革，着力解决群众合理诉求。认真抓好综治维稳和调解工作，扎实推进禁毒社会化工作，持续开展“五打五整治”、医患矛盾纠纷综合整治等专项行动，争创全国社会管理综合治理先进市，确保社会大局和谐稳定。

各位代表，完成政府各项工作目标和任务，必须加强政府自身建设。我们将坚决做到：服务群众不忘初心。牢固树立“人民利益高于一切”的价值取向，把更多的财力、物力和精力集中到增进民生福祉上来，着力解决群众最关心、最直接、最现实的利益问题，着力构建广覆盖、保基本、多层次、可持续的民生保障网，着力开展“结对帮扶”“一进二访”“志愿服务”等察民情、解民忧活动，打通联系服务群众的“最后一公里”，用真心真情践诺初心、温暖人心、赢得民心！依法用权不越边界。坚持法定职责必须为、法无授权不可为，加快建设法治政府。主动接受人大、政协和全社会的监督，加强行政监察、执法监督和审计监督；严格实施“权力清单”“责任清单”“负面清单”制度，做到行政权力进清单、清单之外无权力，坚决防止公共权力部门化、部门权力个人化，既让权力“进笼子”，又让权力“晒太阳”，确保行政权力始终在法治轨道上运行！敢于担当不辱使命。有多大担当才能干多大事业，尽多大责任才会有多大成就。我们将淡化“唱功”，强化“做功”，不漂浮，不敷衍，不推诿，在加快发展上担当重任，在改善民生上担负重托，在破解难题上担起重担，决不把当前问题留给后人、留给他人。坚决落实中央“三个区分开来”的要求，为敢于担当的干部担当，为敢于负责的干部负责，大力弘扬“想干事、不避事，敢干事、不怕事，干成事、不误事”的良好氛围，推动岳阳各项工作走在前列、多作贡献。清廉从政不碰底线。着力增强“四个意识”，严格落实党风廉洁建设“一岗双责”和廉洁从政有关规定，加强惩治和预防腐败体系建设，持之以恒地反“四风”、转作风、正行风，坚持清白从政、干净干事、严守底线，着力营造干部清正、政府清廉、政治清明的政治生态。

各位代表，同志们！面对“一极三宜”江湖名城建设的壮丽蓝图，唯有一往无前才能扛起使命，唯有砥砺奋进才能再创辉煌。让我们紧密团结在以习近平同志为核心的党中央周围，在中共岳阳市委的坚强领导下，紧紧依靠全市人民，同心协力，奋发求为，努力谱写富饶美丽幸福新湖南的岳阳篇章！
